# Bioresorbable Nonwoven Patches as Taxane Delivery Systems for Prostate Cancer Treatment

**DOI:** 10.3390/pharmaceutics14122835

**Published:** 2022-12-17

**Authors:** Joanna Jaworska, Arkadiusz Orchel, Anna Kaps, Marzena Jaworska-Kik, Anna Hercog, Mateusz Stojko, Jakub Włodarczyk, Monika Musiał-Kulik, Małgorzata Pastusiak, Marcelina Bochenek, Marcin Godzierz, Janusz Kasperczyk

**Affiliations:** 1 Centre of Polymer and Carbon Materials, Polish Academy of Sciences, M. Curie-Sklodowskiej 34, 41-819 Zabrze, Poland; 2 Department of Biopharmacy, Faculty of Pharmaceutical Sciences in Sosnowiec, Medical University of Silesia, Jedności 8, 41-200 Sosnowiec, Poland

**Keywords:** polymeric drug delivery systems, prostate cancer therapy, docetaxel, cabazitaxel

## Abstract

Prostate cancer is the second most common cancer in males. In the case of locally advanced prostate cancer radical prostatectomy is one of the first-line therapy. However, recurrence after resection of the tumor can appear. Drug-eluting bioresorbable implants acting locally in the area of the tumor or the resection margins, that reduce the risk of recurrence would be advantageous. Electrospinning offers many benefits in terms of local delivery so fiber-forming polyesters and polyestercarbonates which are suitable to be drug-loaded were used in the study to obtain CTX or DTX-loaded electrospun patches for local delivery. After a fast verification step, patches based on the blend of poly(glycolide-ε-caprolactone) and poly(lactide-glycolide) as well as patches obtained with poly(lactide-glycolide- ε-caprolactone) were chosen for long-term study. After three months, 60% of the drug was released from (PGCL/PLGA) + CTX and it was selected for final, anticancer activity analysis with the use of PC-3 and DU145 cells to establish its therapeutic potential. CTX-loaded patches reduced cell growth to 53% and 31% respectively, as compared to drug-free patches. Extracts from drug-free patches showed excellent biocompatibility with the PC-3 cell line. Cabazitaxel-loaded bioresorbable patches are a promising drug delivery system for prostate cancer therapy.

## 1. Introduction

Prostate cancer is the second most common cancer in males. WHO denoted that in 2020 there were 1.41 mln new cases of prostate cancer [1]. Prostate cancer therapy is different, depending on the stage of the disease or the age of the patient. In the case of high-risk and/or locally advanced prostate cancer, radical prostatectomy and radiation therapy are the first-line therapies. The choice of the method is made by the physician and also by the patient itself. Unfortunately, the treatment outcomes of these high-risk patients are unsatisfactory and prostate cancer-specific mortality is 28.8% and 35.5% after 10 and 15 years, respectively [2]. There is a need to improve these outcomes. Surgery is used mostly for patients whose expected survival is over 10 years and the tumor does not exceed the anatomical limits of the prostate [3]. Surgery involves resection of the prostate gland with seminal vesicles and groups of lymph nodes in the pelvis (frequently performed laparoscopically). Unfortunately, recurrence after excision of the tumor can appear (in the lodge), in regional lymph nodes, or in the form of distant metastases. Drug-eluting bioresorbable implants acting locally in the area of the tumor or in the area of the resection margins that reduce the risk of recurrence would be much more beneficial.

Local delivery guarantees higher local drug concentrations when compared to those obtained with traditional systemic delivery methods. Zoladex is an example of an implant that delivers hormones locally and is used in the case of prostate cancer therapy. It is based on PLGA. All of the commercial products (Lupron Depot, Prostap, Decapeptyl) focused on local delivery, available in prostate cancer therapy, are designed for hormone release.

Electrospinning offers many benefits in terms of local delivery. In spite of the fact that electrospinning is an old concept for fiber fabrication, a large increase in interest in this technique has been noticed lately [4]. It is caused by the possibilities it offers in terms of local drug delivery such as a safe and easy encapsulation technique for the therapeutic cargo, a safe release at the target site, increased local drug concentration due to the large surface area, high volume to mass ratio, reduced toxicity and drug-loading capacity. The bioresorbable polymers based on polyesters and polyestercarbonates are the perfect materials to be chosen for a drug delivery system for local administration in locally advanced prostate cancer treatment. These polymeric materials can be processed by electrospinning (they are very good fiber-forming polymers). Polyesters and polyestercarbonates gained much attention in biomedical applications for decades as drug delivery systems [5,6,7], bioresorbable scaffolds [6], and implantable devices [8,9]. Some of them are approved by the Food and Drug Administration, such as polylactide. Their functionality is mainly based on their ability to degrade since they easily undergo the reaction of hydrolysis [10]. However, this ability to degrade can be widely controlled by modification of the structure which can be conducted during the polymerization reaction [11,12]. Aliphatic polyesters obtained with ring-opening polymerization (ROP) possess good mechanical properties. They are biodegradable and biocompatible. They can be copolymerized with other compounds in order to modify their properties. Copolymerization with carbonate units allows for modifying the polymeric microstructure, mechanical properties, and degradation rate [13]. Polymers containing carbonate units are more flexible and their degradation products exhibit reduced acidity in comparison with aliphatic polyesters which may be important during healing processes after the implantation procedure. Proper control of composition, microstructure, and molecular weight is crucial in view of further medical applications [14]. Electrospun DDS based on polyesters have been already presented in the literature. Jin et al., obtained gelatin/polycaprolactone electrospun fibers loaded with cis-platinum for prostate cancer therapy. However, high initial burst release was detected within the first 1 h and during that time 70% of the drug was released [15].

Paclitaxel together with docetaxel are first-generation taxanes and have been developed into numerous commercialized formulations e.g., paclitaxel formulated with Kolliphor EL (Taxol^®^, Bristol-Myers Squibb, NY, USA), docetaxel formulated in Tween 80 (Taxotere^®^, Sanofi Mature IP, Paris, France), and albumin-bound paclitaxel (Abraxane^®^, Celgene, NY, USA) [16]. Cabazitaxel is a novel second-generation taxane formulated in polymeric micelles with Tween 80 as Jevtana. Cabazitaxel has activity both in docetaxel—sensitive and docetaxel—resistant tumors. [17] Several novel cabazitaxel delivery platforms based on lipid micelles, polymeric micelles, conjugates, and protein-bound nanoparticles have been already presented in the literature [17] e.g., cabazitaxel-loaded PLGA nanoparticles [18]. Electrospun taxane delivery systems are less studied e.g., docetaxel was incorporated into PVA nanofibers [19,20].

The aim of the study was the development and comparison of the docetaxel and cabazitaxel biodegradable delivery systems in a form of electrospun implantable patches. The CTX-DDS and DTX-DDS were obtained with bioresorbable polyester and polyestercarbonates. After initial characterization and fast degradation scan, the anticancer activity of chosen CTX-DDS was studied using PC-3 and DU145 prostate cancer cells. The results confirm the potential of the developed patches for prostate cancer therapy.

## 2. Materials and Methods

### 2.1. Synthesis

Poly(L-lactide-*co*-glycolide) 85/15; PLGA was synthesized in bulk via ring-opening polymerization (ROP) of L-lactide and glycolide (first, at 130 °C for 24 h and next, at 115 °C for 72 h). Copolymerization of poly(glycolide-ε-caprolactone) 10/90; PGCL was carried out in bulk at 120 °C for 72 h in an argon atmosphere. Poly(lactide-trimethylene carbonate) 75/25; PLTMC was prepared by ring-opening polymerization of the L-lactide and trimethylene carbonate monomers. The temperature and time of synthesis was 150 °C and 27 h, respectively.

Terpolymer of poly(lactide-glycolide-ε-caprolactone) 75/10/15; PLGCL was obtained at 120 °C and the time of reaction was 96 h. Zirconium (IV) acetylacetonate Zr(acac)4 (Sigma Aldrich, Poland) was used as an initiator for all polymers. The obtained materials were dissolved in chloroform for purification and precipitated in cold methanol. Finally, the purified polymers were dried in a vacuum at room temperature to constant weight.

Monomers: L-Lactide, glycolide, and trimethylene carbonate were purchased from HUIZHOU Foryou Medical Devices Co., Ltd., Huizhou, China, and ε-caprolactone from ACROS Organics, Darmstadt, Germany.

### 2.2. Preparation of Electrospun Patches

Synthesized polymers were dissolved in CH_2_Cl_2_ (Avantor Performance Materials Poland S.A., Gliwice, Poland) and then mixed with docetaxel or cabazitaxel (LC Laboratories, Woburn, MA, USA). The concentration of each solution prepared for electrospinning was optimized in order to obtain a structure with well-developed fibres. Different nonwovens were obtained with the various amount of the drug: 3–5% (*w*/*w*) (Table 1) using a TL-Pro-BM electrospinning unit (Tong Li Tech, Shenzen, China). The device was equipped with two high voltage power supplies. One of them, that generates positive electrical potential, was applied to the spinneret, while the second one, that generates negative potential, was applied to the fibers collector, in form of a steel mandrel of 27 mm diameter (rotating collector). Polymer solutions were dosed to the spinning nozzle through a PTFE capillary, by using a PHD Ultra 4400 syringe pump (Harvard Apparatus, Holliston, MA, USA). Nonwoven mats were produced according to process parameters, presented in Table 1.

### 2.3. Degradation Study

In vitro degradation study of drug-loaded nonwovens (discs with 1 cm diameter) was conducted in 5 mL of 0.01 M phosphate-buffered saline (PBS, pH 7.4 and 6.8) at 37 °C for 84 days, under continuous agitation at 240 rpm. After sampling at the predetermined intervals, the buffer was replaced to maintain the sink condition. The degradation rate was analyzed on the basis of weight loss [%]. The percentage of weight loss (*W_L_*%) was calculated according to the following equation: $W_{L}\left(\%\right)=\frac{W_{0}-W_{dry}}{W_{0}}\times 100$, where *W*_0_ is the initial weight and *W_dry_* is the residual weight of the materials dried under vacuum until constant weight has been achieved.

### 2.4. In Vitro Drug Release

Drug release was investigated under in vitro conditions at 37 °C in PBS at pH 7.4 (or pH = 6.8) for 84 days. After sampling at the predetermined intervals, the buffer was replaced to maintain the sink condition. The samples were collected for the evaluation of the amount of released drug. High-performance liquid chromatograph (VWR-Hitachi/LaChromElite^®^) equipped with a LiChrospher^®^ RP-18 column (250 mm × 4 mm, 5 μm) and LiChrospher^®^ RP-18 guard column (4 mm × 4 mm, 5 μm) was employed. The mobile phase consisted of acetonitrile and water (60:40, *v*/*v*) and was delivered at a flow rate of 1 mL/min. Docetaxel and cabazitaxel were detected at a wavelength of 227 nm.

### 2.5. Nonwovens Characterization

The morphology of the obtained samples was analyzed using Scanning Electron Microscopy (SEM, Quanta 250 FEG, FEI Company, Hillsboro, OR, USA), operating under low vacuum conditions (80 Pa) and an acceleration voltage of 5 kV from secondary electrons collected by a Large Field Detector.

Water contact angles were measured with a CAM101 goniometer (KSV Instruments) equipped with a camera (resolution 640 × 480 pixels) and an external temperature adapter (Intelligent Digital Controller OMRON5EGN). The volume of the dosing drop of the demineralized water was 2–3 µL. The results of the contact angle measurements are the arithmetic mean calculated on the basis of 20 photos taken during 1 photo/s.

Thermal properties were determined using Differential Scanning Calorimeter (DSC) (DSC Q2000, TA Instruments, New Castle, DE, USA). The instrument was calibrated using high purity indium. The specimens were heated from −80 to 200 °C under a nitrogen atmosphere (flow 50 mL/min) at a heating rate of 20 °C/min. The melting temperature (T_m_) of the polymers was determined from the first heating run as the peak maximum of melting endotherm. The glass transition temperature (T_g_) was determined as the midpoint of heat capacity change of the amorphous sample obtained by quenching from melt to liquid nitrogen.

X-ray diffraction analysis (XRD) measurements were performed using the D8 Advance diffractometer (Bruker AXS, Karlsruhe, Germany) with Cu-Kα cathode (λ = 1.54 Å), using Bragg-Brentano geometry. The scan rate was 1.2°/min with scanning step 0.02° in the range of 2° to 70° 2Θ. Identification of fitting phases was performed using DIFFRAC.EVA program with ICDD PDF#2 database.

Nuclear Magnetic Resonance Spectroscopy (NMR) was employed to describe the chain microstructure and the polymer composition. ^1^H NMR Spectra were recorded with the use of a 600 MHz Bruker Avance II Ultrashield Plus Spectrometer (Bruker, Karlsruhe, Germany). ^1^H NMR spectra were obtained with 64 scans, 11 µs pulse width, and 2.65 s acquisition time. ^13^C NMR spectra (150 MHz) was recorded with 20 480 scans, 9.4 µs pulse width, and 0.9 s acquisition time. DMSO-d6 was used as a deuterated solvent.

### 2.6. In Vitro Anticancer Activity

In vitro anticancer activity of cabazitaxel-loaded electrospun nanofibers was studied using human prostate cancer cell lines, purchased from American Type Culture Collection (ATCC, Manassas, VA, USA). PC-3 cells (ATCC CRL-1435^TM^) were cultured in F-12K medium (Merck Life Science, Poznan, Poland) supplemented with 10% fetal bovine serum (PAN-Biotech, Aidenbach, Germany), 100 U/mL penicillin, and 100 µg/mL streptomycin and 10 mM HEPES (Merck Life Science, Poznan, Poland). DU145 cell line (ATCC HTB-81^TM^) was maintained in Eagle’s Minimum Essential Medium (EMEM; Merck Life Science, Poznan, Poland) containing 10% fetal bovine serum, 100 U/mL penicillin, and 100 µg/mL streptomycin and 10 mM HEPES. The cells were cultured at 37 °C in a humidified atmosphere containing 5% CO_2_.

Fibrous mats were cut into 10 mm diameter circular pieces with a mass of 19 mg. They were sterilized with the use of electron beam irradiation. As our intention was to study the bioactivity of the nanofibrous CBT carrier in the phase of stable drug release, sterile specimens were preincubated in PBS at 37 °C for 7 days. Subsequently, their cytotoxic activity was determined. Cytotoxicity of CBT-loaded mats was compared to pristine electrospun nanofibers, preincubated in PBS in the same way.

Cytotoxicity of electrospun nonwovens was tested using both direct and indirect contact methods. In the first case, cells were seeded into 24-well plates (Corning, Corning, NY, USA) at an initial density of 3 × 10^4^ cells per well in 1 mL of culture medium and incubated for one day to enable cell adhesion. Then, the culture medium was replaced with a fresh one and preincubated specimens were soaked in the medium. Cells growing in standard conditions were used as a negative control, whereas, a medium containing free 10 nM CBT was added to the positive control wells. Cells were cultured in the presence of nanofibers for 72 h. Subsequently, cell density in wells was evaluated using a sulforhodamine B (SRB) assay with the use of the In Vitro Toxicology Assay Kit, Sulforhodamine B Based (Merck Life Science, Poznań, Poland), according to the manufacturer’s instruction (with minor modifications). Briefly: at the end of an incubation period, cells were fixed in 10% trichloroacetic acid solution (at 4 °C), washed with deionized water, and stained with 0.4% SRB solution (in 1.0% acetic acid). Then, the unincorporated stain was washed out (with the use of 1.0% acetic acid solution) and the protein bound stain was solubilized in 0.5 mL of 10 mM tris(hydroxymethyl)aminomethane solution. Then plates were gently shaken until a homogeneous solution was obtained and 200 µL aliquots of SRB solution were transferred to the 96-well clear plate. Absorbance was read at 570 nm and 690 nm (reference wavelength) using the MRX Revelation plate reader (Dynex Technologies, Chantilly, VA, USA). For cytotoxicity evaluation by the indirect contact assay, preincubated nanofibers were extracted in culture media. Specimens were placed in 1 mL of medium and incubated for 72 h. Then, extraction media were used for cell culture. Cells were plated into 96-well plates at the initial density of 5 × 10^3^ cells per well in 100 µL of the appropriate culture medium and allowed to attach and grow for 24 h. Afterwards, the culture medium was replaced with dilution series of extracts. Extracts were diluted with a culture medium and concentrations of 25%, 50%, and 100% (undiluted extracts) were used. Cell growth was evaluated after 72 h of exposure by means of the SRB assay. In cases of high absorbance values, readings at a suboptimal wavelength (492 nm) were performed. Results were statistically analyzed using a one-way ANOVA followed by the Tukey post hoc test. Calculations were carried out using the Statistica 13.1 software (StatSoft, Tulsa, OK, USA).

Cell death was visualized by vital staining with two fluorescent dyes: 2 µM calcein AM causing green fluorescence of living cells and 4 µM ethidium homodimer-1 (EthD-1) conferring red fluorescence to dead cells (with a leaky plasma membrane). Calcein AM is a lipophilic nonfluorescent compound, easily diffusing through the cell membrane. It is converted by intracellular esterases to the intensely fluorescent calcein. The polyanionic calcein becomes trapped in the cell as long as the plasma membrane integrity is retained. On the contrary, EthD-1 enters exclusively cells with the leaky plasma membrane (typically dead cells) and binds to DNA producing bright red fluorescence of cell nuclei. Analysis of the morphology of cells stained with EthD-1 enables the detection of cells presenting apoptotic changes (such as chromatin condensation and fragmentation of the nucleus). DU145 cells were cultured in 24-well plates with glass bottoms (Wuxi NEST Biotechnology, Wuxi, China) at the initial density of 10^5^ cells/well in 1 mL of medium. Staining was conducted using the LIVE/DEAD™ Viability/Cytotoxicity Kit, For Mammalian Cells (Thermo Fisher Scientific, Waltham, MA, USA) according to the manufacturer’s instruction, following 24 h exposure of cells to tested agents. Stained cells were observed under a fluorescence microscope (Nikon Eclipse TS-100F, NIKON, Tokyo, Japan), and photographed using a Nikon DS-Fi1 digital camera.

## 3. Results

### 3.1. Taxane Delivery Systems: Initial Characterization and Fast Degradation Scan

Five different electrospun materials have been obtained loaded with docetaxel or cabazitaxel.

The characterization of nonwovens’ properties has been presented in Table 2. The concentration of each solution prepared for electrospinning was optimized separately in order to obtain nonwovens with well-developed fibrous structures (Table 1). Thus, the concentration of the solutions differed, depending on the kind of polymer.

The drug content has been originally set to 5% (*w*/*w*) however, a different degree of drug incorporation was obtained in the final samples, depending on the polymer. The selected drug content was chosen based on our previous study [21]. Too high drug loading can result in the inhibition of drug release [21].

In the case of bioresorbable materials obtained from thermoplastic polymers that are intended to be implanted in the body, thermal properties are usually analyzed. The values of melting enthalpy ΔH and glass transition temperature T_g_ were determined and presented in Table 2. For (PLTMC/PLGA) + DTX, PLGCL + DTX, and PLGA + DTX patches, the melting enthalpy is very low which suggests an irregular structure of these polymers, opposite to (PGCL/PLGA) + DTX and (PGCL/PLGA) + CTX. Detected Tg values for (PLTMC/PLGA) + DTX, PLGCL + DTX and PLGA + DTX patches are lower than for (PGCL/PLGA) + DTX and (PGCL/PLGA) + CTX. T_g_ value has an influence on the materials’ behaviour after implantation. At temperatures above T_g_, the amorphous polymer acts similar to rubber, and below T_g_, similar to a glass. When polymer has a T_g_ around body temperature it may be more ductile after implantation in comparison with room temperature [22].

According to XRD analysis samples: (PLTMC/PLGA) + DTX, PLGCL + DTX, and PLGA + DTX show amorphous nature, while samples: (PGCL/PLGA) + DTX and (PGCL/PLGA) + CTX exhibit crystalline peaks (results correspond with the DSC analysis). These peaks were identified as an orthorhombic unit cell (P2_1_2_1_2_1_ structure) of poly(caprolactone), according to ICCD PDF#2 database, card No. 00-062-1286. No evident peaks typical for DTX were detected in all drug-loaded samples, which suggests good molecular dispersion of the drug. It is also worth mentioning that no guest-host interactions were detected (Figure S1).

Materials based on PGCL/PLGA blend are highly hydrophobic, especially (PGCL/PLGA) + CTX. Among these two materials, (PGCL/PLGA) + DTX is characterized with a lower contact angle. Other materials are hydrophilic (Figure S2).

**Figure 1:**
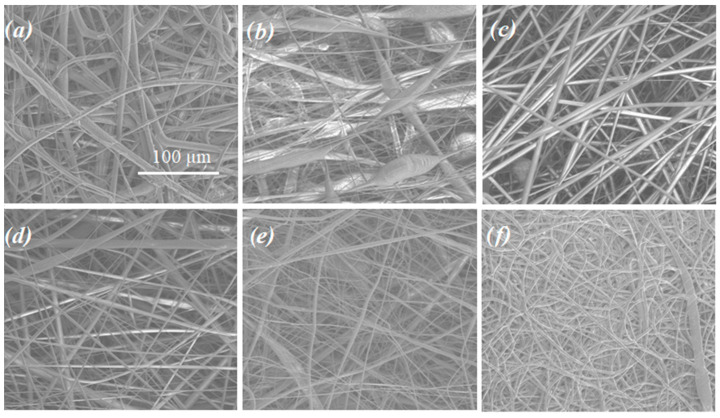
SEM micrographs of drug-loaded nonwoven patches obtained by electrospinning; (**a**) (PGCL/PLGA) + CTX, (**b**) (PGCL/PLGA) + DTX, (**c**) (PLTMC/PLGA) + DTX, (**d**) PLGCL + DTX, (**e**) PLGA + DTX, (**f**) drug- free PGCL/PLGA.

The morphology of the nonwoven patches is presented in Figure 1. Very well-developed fibers were obtained in the case of (PLTMC/PLGA) + DTX, PLGCL + DTX as well as (PGCL/PLGA) + CTX. The morphology of the (PGCL/PLGA) + CTX and (PGCL/PLGA) + DTX fibers differs, in spite of using the same polymeric material (the same ratio of the blend components 1/1 and the same concentration of the solution prepared for the electrospinning). The influence of the drug on the materials’ morphology is observed in that case. In the case of (PGCL/PLGA) + DTX, beads are observed. Beads formed during electrospinning might influence uneven drug release so our purpose was to obtain as uniform fibers as possible.

**Figure 2:**
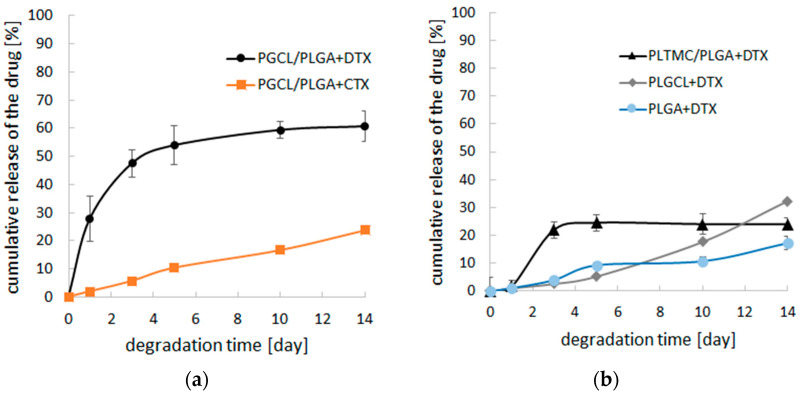
Cumulative release of drug-loaded patches during 14 days of incubation in PBS pH = 7.4 (n = 3); (**a**) (PGCL/PLGA) + DTX and (PGCL/PLGA) + CTX patches; (**b**) (PLTMC/PLGA) + DTX, PLGCL + DTX and PLGA + DTX patches.

The most important feature of designed degradable patches for prostate cancer therapy is to provide sustained release of the taxane, without rapid release in the initial and longer period. Thus, the prepared drug delivery systems have been degraded for 14 days in order to evaluate the usefulness of obtained systems. For (PGCL/PLGA) + DTX, nearly 30% of the drug was released during the first day (Figure 2a). In the case of (PLTMC/PLGA) + DTX, the suspension of the drug release was noted after the third day which is inappropriate for chemotherapy (Figure 2b). For the other materials, slow release of the drug in the initial days was obtained which is beneficial in view of anticancer therapy

**Figure 3:**
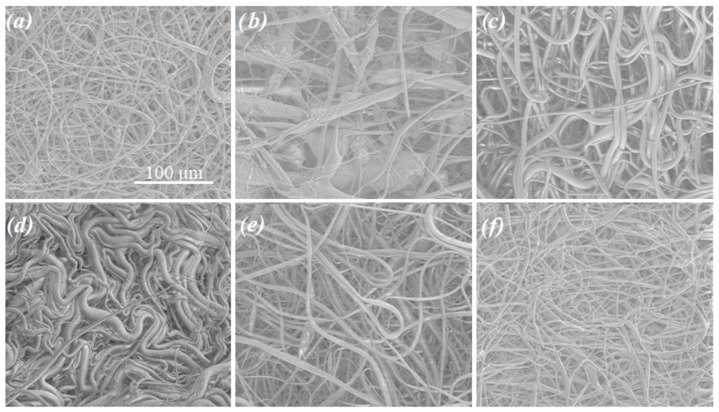
SEM micrographs of drug-loaded nonwoven patches; (**a**) PGCL/PLGA + CTX, (**b**) (PGCL/PLGA) + DTX, (**c**) (PLTMC/PLGA) + DTX, (**d**) PLGCL + DTX, (**e**) PLGA + DTX, (**f**) drug- free PGCL/PLGA after fast degradation scan (14 days of incubation in PBS pH = 7.4 and 37 °C).

The second factor which was important in the initial verification of the material’s usefulness was the morphology of the fibrous patches and their changes during degradation. Figure 3 presents the changes after 14 days of incubation in PBS, pH = 7.4. The fibrous structure is observed for every patch. However, in the case of PLGCL + DTX, fibers start to lose their initial structure, they merge and connect with each other into a compact structure. The entanglement seen in Figure 3 results from the degradation of the material.

On the basis of a 14-day degradation scan and drug release (PGCL/PLGA) + CTX and PLGCL + DTX patches were selected for further long-term verification.

### 3.2. Taxane Delivery Systems: Long-Term Drug Release Study and Degradation Profile

**Figure 4:**
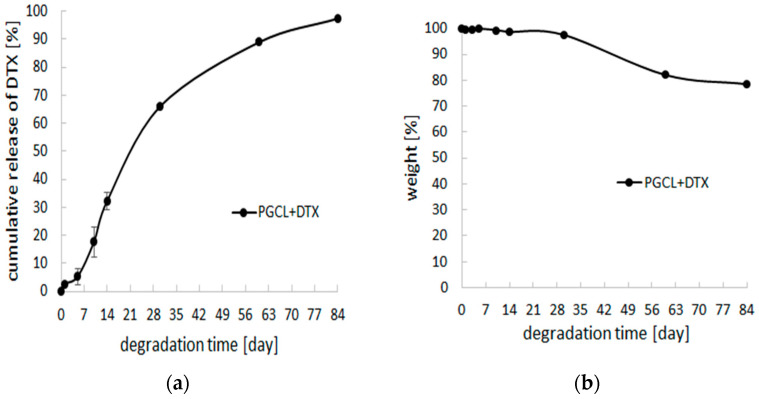
(**a**) cumulative release of DTX-loaded patches, (PLGCL) + DTX, during 84 days of incubation in PBS, pH = 7.4, (n = 3); (**b**) weight changes during degradation of (PLGCL) + DTX patches in PBS, pH = 7.4, (n = 3).

In the case of PLGCL + DTX patches, long-term analysis did not confirm their usefulness for prostate cancer therapy. Although in the first period of time, the drug release seemed to be appropriate for delivering an anticancer drug, in the longer period of time—the course of the drug release process changed unfavorably (Figure 4).

**Figure 5:**
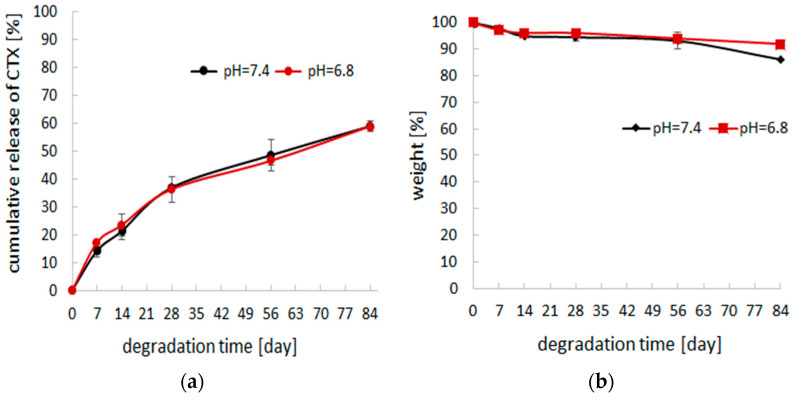
(**a**) cumulative release of CTX-loaded patches, (PGCL/PLGA) + CTX, during 84 days of incubation in PBS (pH = 7.4 and pH = 6.8), (n = 3); (**b**) weight changes during degradation of PGCL/PLGA) + CTX patches in PBS (pH = 7.4 and pH = 6.8), (n = 3).

In the case of (PGCL/PLGA) + CTX, the course of drug release was more even (Figure 5). According to this, the drug delivery was also analysed in the PBS with a lower value of pH (pH = 6.8) and compared with release in pH = 7.4. Changes were insignificant.

**Figure 6:**
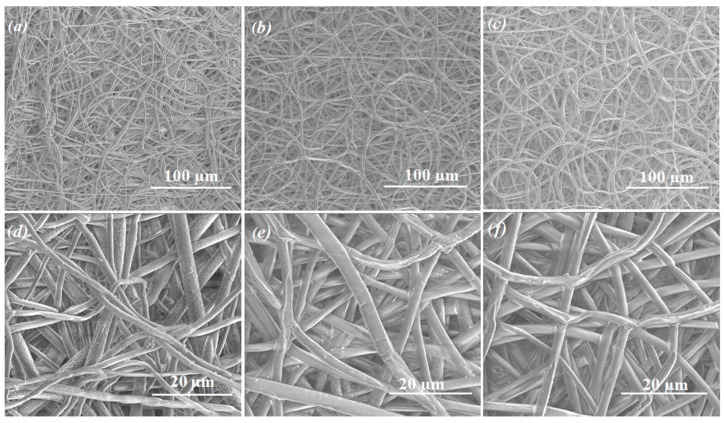
SEM micrographs of PGCL/PLGA + CTX patches during incubation in PBS pH = 7.4 and 37 °C. (**a**,**d**) 4 weeks; (**b**,**e**) 8 weeks; (**c**,**f**) 12 weeks of incubation.

Moreover, the changes in the fibers’ morphology during the degradation of the drug delivery system were beneficial in the case of (PGCL/PLGA) + CTX. The material maintained its fibrous structure during 3 months of incubation in the degradation medium (Figure 6). Detailed analysis of the changes of the fibers morphology of (PGCL/PLGA) + CTX was presentedin Figure S3.

(PGCL/PLGA) + CTX patches were found to be more suitable for prostate cancer therapy and were selected for final, anticancer activity analysis.

For selected (PGCL/PLGA) + CTX patches, the mechanism of the release of the drug was analyzed. To evaluate the release kinetics of CTX from electrospun patches, the release data points were subjected to zero order and first order mathematical models as well as the Higuchi model and Korsmeyer-Peppas model. The R2 was chosen to assess the approximation accuracy. Table S3 presents values of the correlation coefficient calculated from different mathematical release models.

### 3.3. Cabazitaxel-Loaded PGCL/PLGA Patches: The Microstructure of the Polymeric Chain

**Figure 7:**
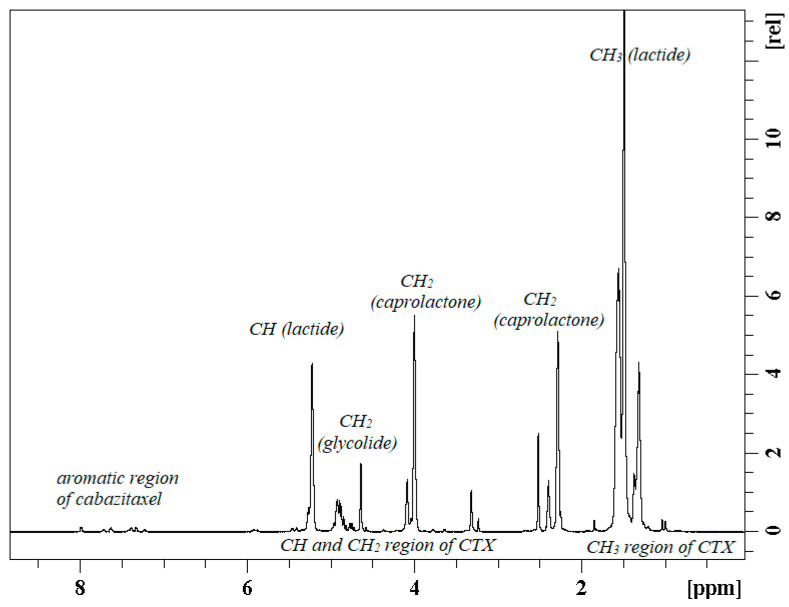
^1^H NMR spectrum of (PGCL/PLGA) + CTX patches in DMSO-d6.

**Figure 8:**
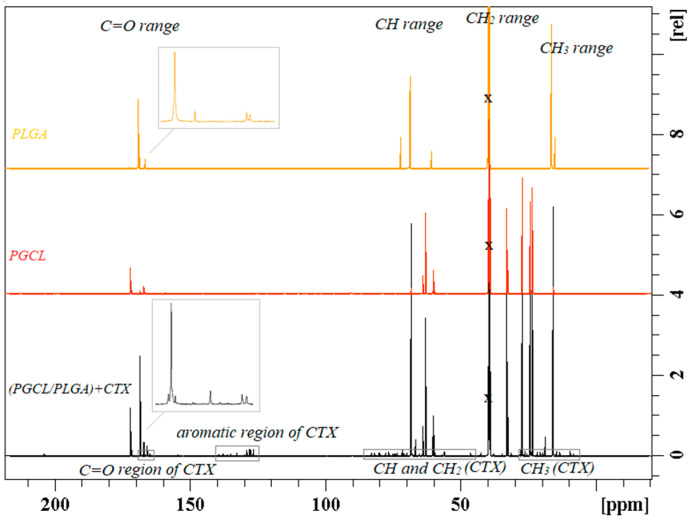
^13^C NMR spectrum of (PGCL/PLGA) + CTX patches in DMSO-d6 with the description of the spectral range of main functional groups in the polymer chain (descriptions above the spectra) and assignment of the peaks associated with cabazitaxel (descriptions in the bottom of the spectra).

**Figure 9:**
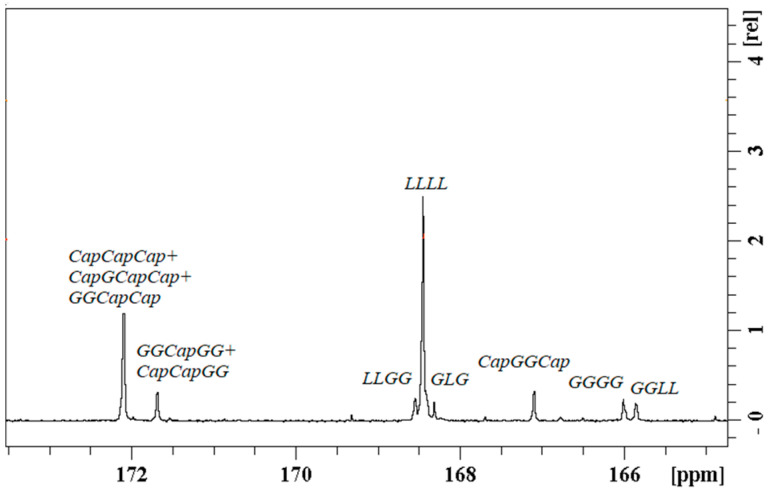
Carbonyl carbon range (C=O) of the ^13^C NMR spectrum of (PGCL/PLGA) + CTX. Signals are described according to the nomenclature presented in previous publications [23,24].

Selected (PGCL/PLGA) + CTX patches were additionally characterized by Nuclear Magnetic Resonance Spectroscopy (Figure 7, Figure 8 and Figure 9). ^13^C NMR spectra were used to characterize the microstructure of the polymeric chain [12,23] (the average length of blocks, and the randomness of the polymer chain). (PGCL/PLGA) + CTX were prepared by mixing separate copolymers PGCL and PLGA and the blend was obtained. Structural sequences built from glycolidyl units (they are created from glycolide monomer which in the final polymer chain can be placed in long blocks or in short blocks that are surrounded by other comonomers) can be easily calculated from the spectral range of carbonyl carbon in the ^13^C NMR spectrum and the value of the average length of blocks can be obtained (Figure 8 and Figure 9). This is helpful in describing the chain of the polymer. Values that characterize the polymer chain were analysed for separate copolymers. The average length of glycolidyl blocks l_GG_ (created from glycolide monomer) in PGCL was 0.5 and the average length of caproyl blocks l_Cap_ (created from caprolactone monomer) was 4.7. The degree of randomness, R, was 1.18. In the case of PLGA, the l_GG_ was 2.2 and l_LL_ = 10 (lactidyl blocks created from lactide monomer), and the degree of randomness was 0.35.

In the case of the blend, the above calculations are limited so only the comparison of the spectra was provided in Figure 8.

### 3.4. In Vitro Anticancer Activity of Cabazitaxel-Loaded PGCL/PLGA Patches

**Figure 10:**
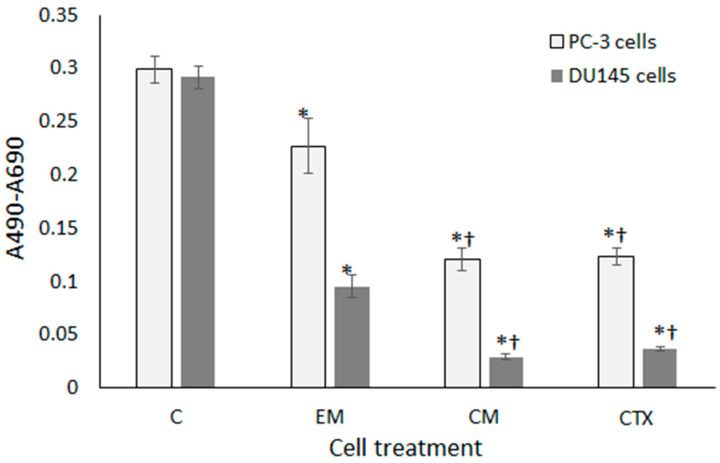
Growth of prostate cancer cells in the presence of nanofibers (direct contact), assessed using the SRB assay; (C) control; (EM) drug-free mat; (CM) CTX-loaded mat; (CTX) 10 nM CTX solution. Cells were incubated with patches for 3 days; results are shown as mean ± SD; * *p* < 0.05 compared to control; † *p* < 0.05 compared to the empty mat (ANOVA).

The cytotoxic effects of CTX-loaded PGCL/PLGA patches placed in direct contact with prostate cancer cells, determined using sulforhodamine B staining, are illustrated in Figure 10.

**Figure 11:**
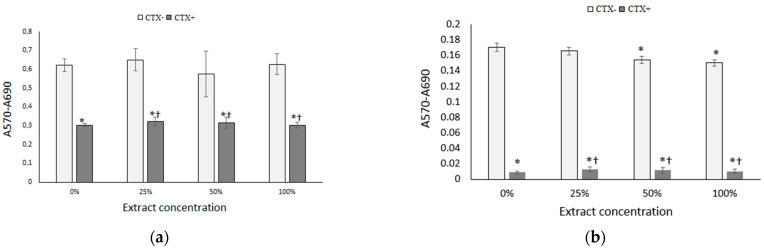
(**a**) Growth of PC-3 cells; and (**b**) growth of DU145 cells in the presence of various concentrations of extracts of nonwovens (indirect contact), assessed using the SRB assay. At the extract concentration of 0%, cells designated as CTX+ were treated with the 10 nM CBT solution; * *p* < 0.05 compared to control; † *p* < 0.05 compared to the empty mat (ANOVA).

Both cell lines proliferated at a similar rate in control wells. In general, DU145 cells turned out to be more susceptible to the effects of tested agents. In wells with PC-3 and DU145 cells treated with CTX-loaded patches, cell growth was reduced to 53% and 31% respectively, as compared to wells treated with drug-free patches. It means, that the cell growth inhibitory activity of CTX-loaded patches was comparable to the free drug. However, in cultures incubated with drug-free PGCL/PLGA patches, cell proliferation was significantly decreased with respect to control, especially in the DU145 cell line. We assumed, that it could result from mechanical damage of cells by nonwovens or hindered diffusion of oxygen to cells under the mat. To avoid the physical presence of mats in cell culture, we prepared extracts from electrospun materials in culture media and they were tested for cytotoxicity. As shown in Figure 11, extracts from drug-free patches showed excellent biocompatibility with the PC-3 cell line, whereas, in DU145 cells only a slight inhibition of growth was found at extract concentrations of 50% and 100%.

On the other hand, extracts obtained from drug-loaded nanofibers were highly cytotoxic against prostate cancer cells at all the tested concentrations. All the results described above proved the high efficacy of our nonwoven carrier in drug delivery to cancer cells at a stage of stable drug release.

**Figure 12:**
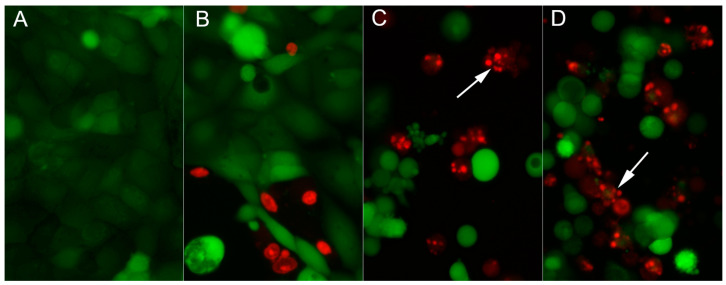
Live/Dead staining of DU145 cells cultured for 24 h in the presence of nanofibers; (**A**) control; (**B**) drug-free patches; (**C**) CTX- loaded patches; (**D**) 10 nM CBT solution; arrows indicate late apoptotic cells; magnification ×200.

Figure 12 shows control DU145 cells stained with the Live/Dead Viability/Cytotoxicity Kit. Most of the visible cells are attached and spread on the plastic substratum. They exhibit green fluorescence, resulting from the cumulation of calcein within the cytoplasm, indicating their good viability.

In cultures growing in the presence of drug-free patches, in addition to normal appearing cells, there are some dead cells with nuclei stained with EthD-1 (demonstrating orange fluorescence; Figure 12B). Interestingly, these cells did not show apoptotic morphology. We assumed that these cells could be mechanically damaged by fibrous specimens placed in wells or they could die as a result of impeded diffusion of oxygen under the mat. In cell cultures treated with CTX- loaded patches (Figure 12C), there were very few spread, polygonal cells. Instead, there were visible numerous rounded cells, glowing green, some of which underwent shrinkage and fragmentation. Additionally, there were a lot of EthD-1 positive (orange) nuclei presenting morphological alterations typical of apoptosis: chromatin condensation and division into small spherical fragments. It is well known that, in cells undergoing apoptosis, the plasma membrane integrity is maintained for a relatively long time (in contrast to necrotic cell demise) [25]. Therefore, EthD-1 positive cells represent a population at the late stage of programmed cell death. Rounded or fragmented/blebbing cells with green fluorescence most probably represent cells at the early stages of apoptosis. The above-described phenomena occurring in cultures incubated with CTX- loaded patches were very similar to those observed in cells treated with the 10 nM CBT solution (Figure 12D).

## 4. Discussion

Different electrospun docetaxel- or cabazitaxel-loaded patches were obtained in the study using polyesters and polyestercarbonates. Patches were developed to achieve local delivery of the drugs used in prostate cancer therapy nowadays. This type of administration ensures a high local concentration of the incorporated drug with reduced systemic drug levels. Moreover, the fibrous structure of the electrospun carrier offers many benefits in terms of local delivery due to the large specific surface area. Polyesters and polyestercarbonates used in the study are fiber-forming polymers. The kind of polymer was chosen on the basis of our previous study and was aimed to provide regular delivery of the drug and appropriate morphology, adjusted to the prostate cancer therapy. One of the treatments of locally advanced prostate cancer is radical prostatectomy so flexible material for filling the excision lodge, delivering simultaneously the anticancer drug in a regular manner without burst effect would be beneficial. The kind of polymer was chosen partially on the basis of our previously published study, where poly(glycolide-ε-caprolactone) PGCL and poly(lactide-glycolide) PLGA blend (abbreviated as PGCL/PLGA) with different content of the components was obtained [21]. Two kinds of taxane were incorporated into the same material—PGCL/PLGA blend to evaluate the influence of the drug on the properties of the drug delivery system. We also used polymers composed of the same structural units as in the blend such as: lactide, glycolide, and ε-caprolactone, however, they were incorporated in a terpolymer chain of poly(lactide-glycolide-ε-caprolactone) PLGCL. PLGCL terpolymer had an amorphous character which could further ensure appropriate drug release. PLGA was chosen for comparison. The other material chosen as a carrier for the drug was poly(lactide-trimethylene carbonate) PLTMC which was incorporated in the PLTMC/PLGA blend. PGCL, initially set into the PGCL/PLGA blend, was aimed to ensure flexibility due to the caprolactone units. However, PLTMC has a similar characteristic (may ensure flexibility) due to the trimethylenecarbonate units. Moreover, PLTMC’s amorphous character might influence better drug release, than the crystalline character of PGCL. In order to compare the influence of two different components in the PLGA-based blend, PLTMC/PLGA blend was additionally obtained for analysis (as a comparison for PGCL/PLGA blend).

At the initial step, electrospun patches were obtained and characterized during the fast degradation study to check if there is an unfavorable burst effect. After this verification step, patches based on the blend of poly(glycolide-ε-caprolactone) and poly(lactide-glycolide) as well as patches obtained with poly(lactide-glycolide- ε-caprolactone) were chosen for long-term study. They were incubated in PBS during 84 days. During that time, the course of drug release was more even for (PGCL/PLGA) + CTX than for PLGCL + DTX. At the end of the study, 60% of the drug was released from (PGCL/PLGA) + CTX and 97% from PLGCL + DTX patches. In the case of PLGCL + DTX, the course of the drug release was much more intensive. (PGCL/PLGA) + CTX patches were found to be more suitable for prostate cancer therapy. Thus, for selected (PGCL/PLGA) + CTX patches, the mechanism of the release of the drug was analyzed. It appeared that the mechanism is fitted the best to the Higuchi model since the R2 value is close to 1, which in turn indicates that CTX release occurs according to the square root of time but polymer degradation aids also the drug release. Moreover, the changes in the fiber’s morphology were beneficial for selected (PGCL/PLGA) + CTX. The material maintained its fibrous structure during 3 months of incubation in a degradation medium so the benefits connected with nonwoven architecture were provided. Since there is a pH difference between the normal tissues (pH 7.4) and tumor extracellular environment (pH 6.8), (PGCL/PLGA) + CTX patches were additionally incubated in PBS, pH = 6.8 for 3 months, and no significant changes were noted [26]. However, nonwovens presented in the study were designed for the resection margins, where there is no direct contact with massive tumors nevertheless, pH differences might occur.

Singh et al. presented electrospun PVA nanofibers with docetaxel [20]. A burst effect (~31%) was noted in the first hour and during 7 h, 97% of the incorporated drug was released. Similar effects were observed by Attaolahi for DTX-loaded PVA nanofibers with lentinan [19]. Ding et al. obtained electrospun DTX-loaded PLA nonwovens and during 24 days of study 23–30% of the drug was released [27]. However, in the first 24 h, a rapid initial burst was noted. In our study, the DTX release from (PGCL/PLGA) + DTX at the initial period was similar. However, in the case of (PGCL/PLGA) + CTX, where cabazitaxel was loaded, instead of docetaxel, the burst effect was reduced, and only ~2% of the drug was released in this period.

(PGCL/PLGA) + CTX patches were selected for final, anticancer activity analysis. The cytotoxic effects of CTX-loaded patches placed in direct contact with prostate cancer cells were determined using sulforhodamine B staining. In wells with PC-3 and DU145 cells treated with CTX-loaded patches, cell growth was reduced to 53% and 31% respectively, as compared to wells treated with drug-free patches. However, in cultures incubated with drug-free patches, cell proliferation was significantly decreased with respect to control, it could result from mechanical damage of cells by nonwovens. To avoid the physical presence of mats in cell culture, extracts from electrospun materials were prepared in culture media and they were tested for cytotoxicity. Extracts from drug-free patches showed excellent biocompatibility with the PC-3 cell line, whereas, in DU145 cells only a slight inhibition of growth was found at extract concentrations of 50% and 100%. On the other hand, extracts obtained from drug-loaded nanofibers were highly cytotoxic against prostate cancer cells at all the tested concentrations which proves the high efficacy of nonwoven carriers in drug delivery to cancer cells. The results confirm the potential of the developed patches for prostate cancer therapy.

## 5. Conclusions

Different electrospun docetaxel- or cabazitaxel-loaded patches were obtained in the study using aliphatic polyesters and polyestercarbonates. Patches were developed to achieve local delivery of the drugs used in prostate cancer therapy. At the initial step, electrospun patches were obtained and characterized during the fast degradation study to check if there is an unfavourable burst effect. After this verification step, patches based on the blend of poly(glycolide-ε-caprolactone) and poly(lactide-coglycolide) as well as patches obtained with poly(lactide-glycolide- ε-caprolactone) were chosen for long-term study. During a long-term study, 60% of the incorporated drug-cabazitaxel was released and the morphology of the nonwoven structure was maintained. The therapeutic potential was analysed for selected PGCL/PLGA +CTX patches using PC-3 and DU145 prostate cancer cells. The results confirm that cabazitaxel-loaded bioresorbable patches are promising drug delivery systems for prostate cancer therapy.

## Figures and Tables

**Table 1:** Parameters of the electrospinning process.

Title 1	(PGCL/PLGA) + CTX	(PGCL/PLGA) + DTX	(PLTMC/PLGA) + DTX	PGCL + DTX	PLGA + DTX
Concentration of the solution [%] [*w*/*w*]	12 _(PGCL)/_ /12 _(PLGA)_	12 _(PGCL)_ /12 _(PLGA)_	27 _(PLTMC)_ /8 _(PLGA)_	20	12
Positive voltage [kV]	17.0	20.0	20.0	15.0	15.0
Negative voltage [kV]	−3.0	−5.0	−5.0	−4.0	−4.0
TCD ^1^ [cm]	18.0	20.0	20.0	16.5	17.0
Feed rate [mL/h]	3.0	2.5	2.5	2.0	1.5
Needle gauge ^2^	G20	G20	G20	G21	G20
Volume of the solution [mL]	17.0	14.0	18.0	19.0	16.0

^1^ TCD—distance between needle tip and the collector; ^2^ Needle gauge—needle diameter [G].

**Table 2:** Characterization of the patches obtained in the study.

Patch	(PGCL/PLGA) + CTX	(PGCL/PLGA) + DTX	(PLTMC/PLGA) + DTX	PLGCL + DTX	PLGA + DTX
Drug content [%] ^a^	5	3	3	3.7	3.3
Average weight of the patch with 1 cm diameter [mg]	20	17	18.5	11.6	6.3
ΔH ^b^	23.2	28.7	5.8	5.1	4.5
T_g_ [°C] ^c^	−45.4/58.9 *	−42.4/60.9 *	47.9	44.7	45.9

^a^ on the basis of HPLC, ^b^ on the basis of DSC (I run), ^c^ on the basis of DSC (II run), * two values of Tg origins from separate glass transition temperatures detected on DSC curves for (PGCL/PLGA) + CTX and (PGCL/PLGA) + DTX.

## Data Availability

The data that support the findings of this study are available from the corresponding author upon reasonable request.

## Supplementary Materials

The following supporting information can be downloaded at: https://www.mdpi.com/article/10.3390/pharmaceutics14122835/s1, Figure S1. XRD pattern of the drug-loaded patches. Table S1. Water contact angle measurement. Figure S2. Water contact angle for obtained drug-loaded patches. Figure S3. The measured fibers diameter and distribution for drug-loaded patches. Table S2. The average diameter of the pores of obtained drug-loaded patches. Figure S4. The measured fibers diameter and distribution for (PGCL/PLGA) + CTX patches after 12 weeks of degradation. Table S3. Correlation coefficients obtained for (PGCL/PLGA) + CTX patches.
