# Supplementary material for: Bioresorbable Nonwoven Patches as Taxane Delivery Systems for Prostate Cancer Treatment
Source: Pharmaceutics. 2022 Dec 17;14(12):2835. doi: 10.3390/pharmaceutics14122835 (PMC9786168; doi:10.3390/pharmaceutics14122835)
Supplement: Supplementary file 1 [file pharmaceutics-14-02835-s001.zip › pharmaceutics-2029275-supplementary.pdf]

SUPPLEMENTARY MATERIAL

1) XRD measurements

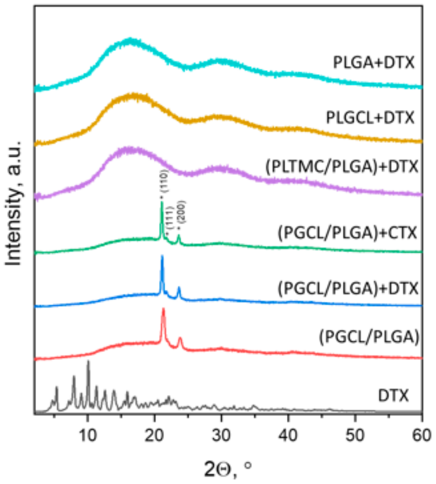

Figure S1. XRD pattern of the drug-loaded patches.

2) Water contact angle measurements

In order to characterize surface properties, water contact angle was measured for 5 materials (drug-loaded patches) presented in the manuscript. Due to the fibrous structure and the character of the nonwoven material it was hard to conduct a measurement in same cases. However, the value of the water contact angle was measured and in the case of particular materials, the surface tension were additionally characterized.

Table S1 Water contact angle measurements

material	water contact angle [°]	surface tension [mN*m^{-1}]
PGCL/PLGA	111.81	86.59
PGCL/PLGA+CTX	118.63	70.49
(PGCL/PLGA)+DTX	96.13	*
(PLTMC/PLGA)+DTX	87.26	59.49
PLGCL+DTX	82.80	62.40
PLGA+DTX	64.13	*

*unable to measure

Materials based on PGCL/PLGA blends are highly hydrophobic, especially PGCL/PLGA+CTX. Among these two materials (PGCL/PLGA)+DTX is characterized with lower water contact angle. It corresponds with the behaviour during drug release. The release of CTX from (PGCL/PLGA)+CTX was slow in comparison with DTX (there is fast release at the beginning of the degradation in the case of (PGCL/PLGA)+DTX).

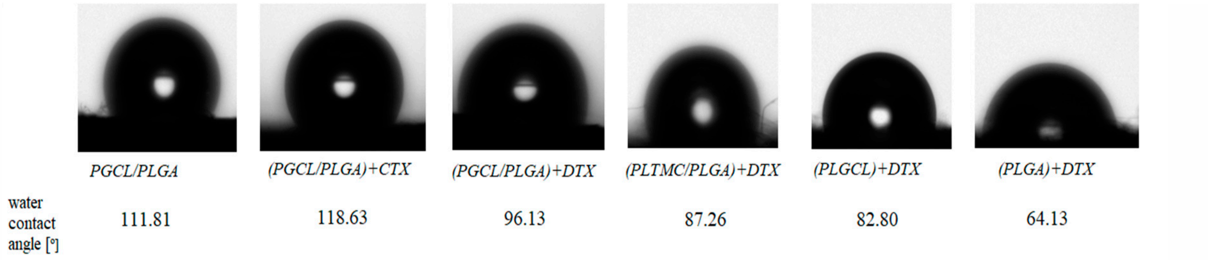

Figure S2. Water contact angle for obtained drug-loaded patches

3) SEM analysis

Detailed SEM analysis was conducted and the fibers diameter and distribution was described. Different sizes of the fibers diameters were observed, depending on the sample. In the case of PLGA+DTX the smallest value was obtained and the biggest for (PLTMC/PLGA)+DTX. In the case of (PLTMC/PLGA)+DTX patches, developed fibers were highly uniform and smooth.

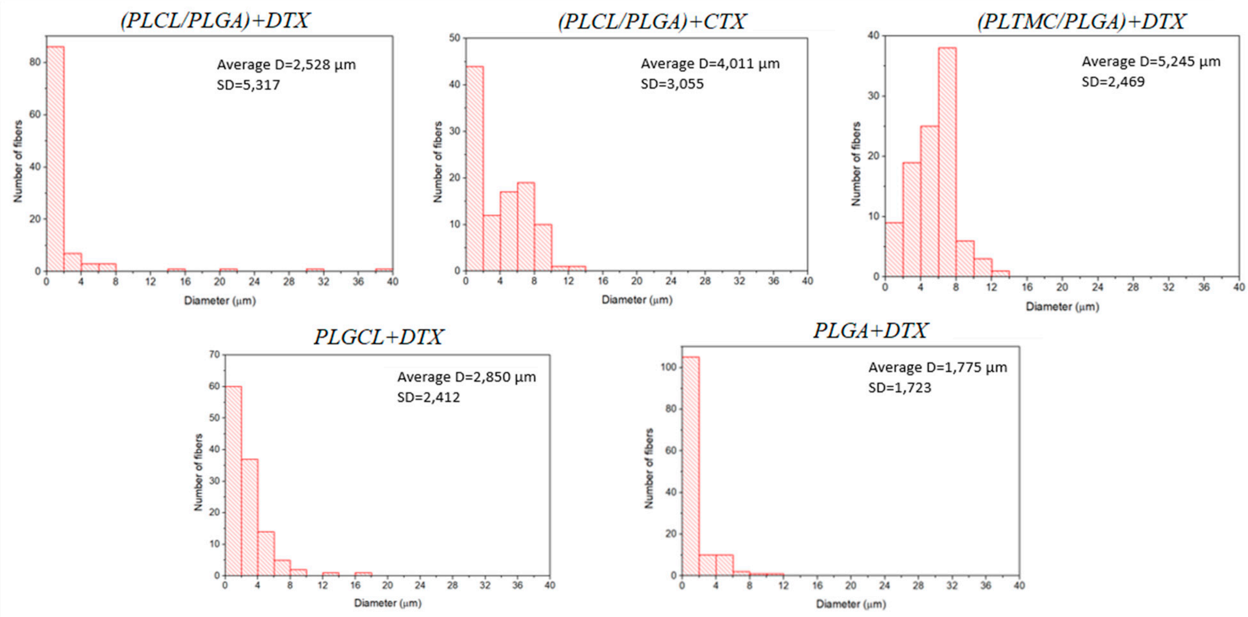

Figure S3. The measured fibers diameter and distribution for drug-loaded patches.

As it comes to the porosity, different sizes of the pores in the inner structure of the nonwoven were measured on the basis of SEM images:

Table S2. The average diameter of the pores of obtained drug-loaded patches

material	the average diameter of the pores
PGCL/PLGA+CTX	10.12 µm
(PGCL/PLGA)+DTX	10.89 µm
(PLTMC/PLGA)+DTX	13.62 µm
PLGCL+DTX	12.28 µm
PLGA+DTX	8.60 µm

In the case of PLGA+DTX the smallest value was obtained and the biggest for (PLTMC/PLGA)+DTX.

4) Changes of the fibers morphology during degradation of (PGCL/PLGA)+CTX

(PGCL/PLGA)+CTX was chosen for long-term study so the detailed analysis of the fibrous structure was conducted for this material. During degradation of (PGCL/PLGA)+CTX, changes of fibers morphology was analysed. In the case of the initial samples of (PGCL/PLGA)+CTX, differences in the fibers morphology were observed. These differences can be seen in the supplementary figure 3. However, during degradation, fibers became smooth and uniform what can be observed in the supplementary figure 4. The fibers diameter decreased from 4.011µm (for initial sample) to 2.912 µm (for sample after 12 weeks of degradation). The SD value also decreased from ~3 to ~0.6.

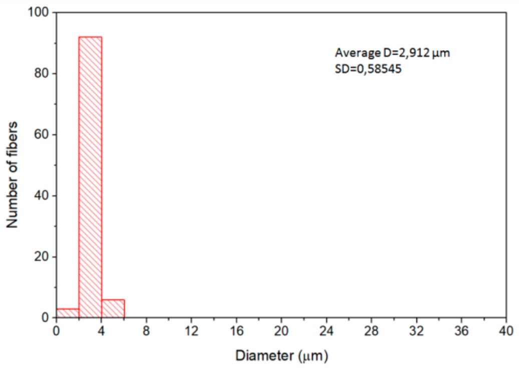

Figure S4. The measured fibers diameter and distribution for (PGCL/PLGA)+CTX patches after 12 weeks of degradation.

5) Correlation coefficients calculated from different mathematical release models

Table S3 Correlation coefficients obtained for (PGCL/PLGA)+CTX patches

**(PGCL/PLGA)+CTX**	R^2
Zero order release model	0.9126
First order release model	0.9681
**Higuchi model**	**0.9917**
Korsmeyer–Peppas model	0.9311
